# Supplementary material for: A systematic review of risk communication in clinical trials: How does it influence decisions to participate and what are the best methods to improve understanding in a trial context?
Source: PLoS One. 2020 Nov 16;15(11):e0242239. doi: 10.1371/journal.pone.0242239 (PMC7668608; doi:10.1371/journal.pone.0242239)
Supplement: S1 Appendix — (DOCX) [file pone.0242239.s002.docx]

S1 Appendix: Search Strategy MEDLINE

1 Risk Assessment/ (682179)

2 Risk Factors/ (1302487)

3 Risk/ (617231)

4 Risk Reduction Behavior/ use ppez (11208)

5 Risk reduction/ use emez (85731)

6 Risk perception/ use psyc2,psyc3,psyc4,psyc5,psyc6,psyc7,psyc8,psyc9,psyc10,psyc11,psyc12,psyc13 (5575)

7 Probability/ (139502)

8 Uncertainty/ use psyc2,psyc3,psyc4,psyc5,psyc6,psyc7,psyc8,psyc9,psyc10,psyc11,psyc12,psyc13 (6873)

9 Probability Judgment/ use psyc2,psyc3,psyc4,psyc5,psyc6,psyc7,psyc8,psyc9,psyc10,psyc11,psyc12,psyc13 (1689)

10 (risk? adj7 (Information or intervention$ or benefit$ or harm$ or adverse effect$ or side effect$ or drug$ or procedure$ or surgery or surgical or treatment?)).tw. (715998)

11 (harm$ adj7 (drug$ or procedure$ or surgery or surgical or treatment?)).tw. (28243)

12 risk.kf. or risk.kw. use emez (205954)

13 or/1-12 (3169283)

14 communication/ use ppez,psyc2,psyc3,psyc4,psyc5,psyc6,psyc7,psyc8,psyc9,psyc10,psyc11,psyc12,psyc13 (99065)

15 communication barriers/ use ppez,psyc2,psyc3,psyc4,psyc5,psyc6,psyc7,psyc8,psyc9,psyc10,psyc11,psyc12,psyc13 (6492)

16 Written communication/ use psyc2,psyc3,psyc4,psyc5,psyc6,psyc7,psyc8,psyc9,psyc10,psyc11,psyc12,psyc13 (7019)

17 health communication/ use ppez (1465)

18 information dissemination/ (34550)

19 medical information/ use emez (64886)

20 Pamphlets/ use ppez (3773)

21 audiovisual aids/ use ppez,emez (7389)

22 educational audiovisual aids/ use psyc2,psyc3,psyc4,psyc5,psyc6,psyc7,psyc8,psyc9,psyc10,psyc11,psyc12,psyc13 (527)

23 medical illustration/ use ppez,emez (8239)

24 Teaching Materials/ (82997)

25 Knowledge Transfer/ use psyc2,psyc3,psyc4,psyc5,psyc6,psyc7,psyc8,psyc9,psyc10,psyc11,psyc12,psyc13 (2534)

26 ((communicat$ or disseminat$) adj3 (patient$ or subject$ or participant$ or trial$)).tw. (67004)

27 ((inform$ or communicat$ or disseminat$) adj3 (intevention$ or benefit$ or harm$ or adverse effect$ or side effect$ or drug$ or procedure$ or surgery or surgical)).tw. (63788)

28 (inform$ adj3 (patient$ or subject$ or participant$ or trial$)).tw. (190186)

29 interactive health communication$.tw. (170)

30 (computer$ adj2 decision mak$).tw. (577)

31 (interacti$ adj3 (aid? or tool$)).tw. (7000)

32 "Patient Acceptance of Health Care"/ use ppez (40647)

33 "Attitude to Health"/ use emez (97939)

34 Client Attitudes/ use psyc2,psyc3,psyc4,psyc5,psyc6,psyc7,psyc8,psyc9,psyc10,psyc11,psyc12,psyc13 (15188)

35 decision support techniques/ use ppez (18491)

36 decision support system/ use emez,psyc2,psyc3,psyc4,psyc5,psyc6,psyc7,psyc8,psyc9,psyc10,psyc11,psyc12,psyc13 (21270)

37 decision making/ or choice behavior/ (379204)

38 decision trees/ use ppez,emez (19028)

39 ((decision$ or decid$) adj3 (support$ or aid$ or tool$ or instrument$ or technolog$ or technique$ or system$ or program$ or algorithm$ or process$ or method$ or intervention$ or material$)).tw. (169941)

40 ((recruit$ or enrol$) adj3 (strateg$ or factor? or method?)).tw. (82412)

41 decision.kf. or decision.kw. use emez (41560)

42 or/14-41 (1339742)

43 13 and 42 (155370)

44 ((risk or probabilit$ or uncertain$) adj3 (inform$ or disseminat$ or communicat$)).tw. (43943)

45 43 or 44 (189736)

46 research subjects/ use ppez,emez (11728)

47 experimental subjects/ use psyc2,psyc3,psyc4,psyc5,psyc6,psyc7,psyc8,psyc9,psyc10,psyc11,psyc12,psyc13 (3783)

48 patient participation/ use ppez,emez (45977)

49 refusal to participate/ use ppez,emez (1704)

50 client participation/ use psyc2,psyc3,psyc4,psyc5,psyc6,psyc7,psyc8,psyc9,psyc10,psyc11,psyc12,psyc13 (0)

51 participation/ use psyc2,psyc3,psyc4,psyc5,psyc6,psyc7,psyc8,psyc9,psyc10,psyc11,psyc12,psyc13 (7238)

52 informed consent/ (128046)

53 (randomi?ed adj3 trial?).tw. (706365)

54 randomized controlled trial.pt. (497697)

55 randomized controlled trial/ use emez (475152)

56 clinical trials/ use psyc2,psyc3,psyc4,psyc5,psyc6,psyc7,psyc8,psyc9,psyc10,psyc11,psyc12,psyc13 (10607)

57 Randomized Controlled Trials as Topic/ use ppez (121999)

58 "randomized controlled trial (topic)"/ (138389)

59 (48 or 49 or 50 or 51 or 52) and (53 or 54 or 55 or 56 or 57 or 58) (11128)

60 ((participa$ or tak$ part or enrol$ or recruit$ or refus$ or decline$ or join$ or enter$) adj5 (trial? or rct or research)).tw. (237878)

61 (participation or recruitment).kf. (5425)

62 (participation or recruitment).kw. use emez (11766)

63 46 or 47 or 59 or 60 or 61 or 62 (271469)

64 45 and 63 (6080)

65 limit 64 to yr="1980 - 2017" (6046) [Embase 3315 MEDLINE 2228 PsycINFO 503]

66 remove duplicates from 65 (4248)
