# Supplementary material for: A systematic review of risk communication in clinical trials: How does it influence decisions to participate and what are the best methods to improve understanding in a trial context?
Source: PLoS One. 2020 Nov 16;15(11):e0242239. doi: 10.1371/journal.pone.0242239 (PMC7668608; doi:10.1371/journal.pone.0242239)
Supplement: Attachment — Submitted filename: PONED1919791_Response_to_reviewers_121219.docx [file pone.0242239.s003.docx]

| **Title** | **A systematic review of risk communication in clinical trials: how does it influence decisions to participate and what are the best methods to improve understanding in a trial context?** |
| --- | --- |
| **Authors** | Maeve Coyle and Katie Gillies |
| **Manuscript ID** | PONE-D-19-19791 |
|  |  |

Dear Editor,

Thank you very much for considering our manuscript for publication in PLOS ONE.

As requested please find below a list addressing the Editorial Comment’s and each point raised by the reviewers. We very much thank the reviewers for their considered responses and we believe that in addressing these we have significantly strengthened the manuscript.

I look forward to hearing from you.

Best wishes

Katie Gillies (on behalf of the authors)

| **Editorial Comments** | | |
| --- | --- | --- |
| **1** | Please ensure that your manuscript meets PLOS ONE's style requirements, including those for file naming. The PLOS ONE style templates can be found at  <http://www.journals.plos.org/plosone/s/file?id=wjVg/PLOSOne_formatting_sample_main_body.pdf> and <http://www.journals.plos.org/plosone/s/file?id=ba62/PLOSOne_formatting_sample_title_authors_affiliations.pdf>  **Response**  Edited accordingly | |
| **2** | Please include your tables as part of your main manuscript and remove the individual files. Please note that supplementary tables (should remain/ be uploaded) as separate "supporting information" files  **Response**  Amended as required and uploaded as a separate document | |
| **3** | In your Data Availability statement, you have not specified where the minimal data set underlying the results described in your manuscript can be found. PLOS defines a study's minimal data set as the underlying data used to reach the conclusions drawn in the manuscript and any additional data required to replicate the reported study findings in their entirety. All PLOS journals require that the minimal data set be made fully available. For more information about our data policy, please see <http://journals.plos.org/plosone/s/data-availability>.  Upon re-submitting your revised manuscript, please upload your study’s minimal underlying data set as either Supporting Information files or to a stable, public repository and include the relevant URLs, DOIs, or accession numbers within your revised cover letter. For a list of acceptable repositories, please see <http://journals.plos.org/plosone/s/data-availability#loc-recommended-repositories>. Any potentially identifying patient information must be fully anonymized.  Important: If there are ethical or legal restrictions to sharing your data publicly, please explain these restrictions in detail. Please see our guidelines for more information on what we consider unacceptable restrictions to publicly sharing data: <http://journals.plos.org/plosone/s/data-availability#loc-unacceptable-data-access-restrictions>. Note that it is not acceptable for the authors to be the sole named individuals responsible for ensuring data access.  We will update your Data Availability statement to reflect the information you provide in your cover letter.  **Response**  Given this is a systematic review, all of the data within the paper is already publicly available online through journal websites. All included studies, and as such the minimal data set, are cited in the reference section. We have provided the DOIs for each of the included studies:  Reference 22 - DOI: [10.1177/009286150604000302](https://doi.org/10.1177%2F009286150604000302)  Reference 23 - DOI: [10.1542/peds.2009-1796](https://dx.doi.org/10.1542%2Fpeds.2009-1796)  Reference 24 - DOI: [10.1177/1740774515585120](https://doi.org/10.1177%2F1740774515585120)  Reference 25 - DOI: [10.1017/S1357530902000558](https://doi.org/10.1017/S1357530902000558)  Reference 26 - DOI: [10.1097/00000539-200302000-00037](https://doi.org/10.1097/00000539-200302000-00037)  Reference 27 - DOI: [10.1186/1472-6947-10-55](https://doi.org/10.1186/1472-6947-10-55)  Reference 28 - DOI: 10.1177/014107689008300710 | |
| **4** | Please amend the file type of your PRISMA checklist to a 'Supporting Information' file type.  **Response**  Edited accordingly | |
| **Reviewer #1:** | | |
| **5** | The authors conduct a systematic review of studies examining different methods of presenting risk and benefit information in the context of clinical trial consents. While the initial search generated a large number of prospective papers, the inclusion criteria, which specifically required the studies to be examining communication in a clinical trial context, resulted in only 7 widely varying papers (all of which relate to hypothetical clinical trials decisions) being actually examined. The authors summarize the findings of these 7 papers and provide some additional context in the discussion.  While the basic structure and writing of the paper were acceptable, I have a number of conceptual concerns.  First, this paper is really more of a review of a systematic review. Yes, there was a systematic process for identifying papers and abstracting information. But, the papers are themselves so varied that no conclusions can be drawn except at the individual study level. In other words, no new insights are gained by the set of information beyond the understanding of best practices that existed previously. Given that this journal explicitly excludes review papers, I am thus skeptical that this paper is appropriate for publication here.  **Response**  The guidance on the PLOS website for Systematic reviews states the following:  **‘Systematic reviews and meta-analyses**  A systematic review paper, as defined by [The Cochrane Collaboration](http://www.cochrane.org/), is a review of a clearly formulated question that uses explicit, systematic methods to identify, select, and critically appraise relevant research, and to collect and analyze data from the studies that are included in the review. These reviews differ substantially from narrative-based reviews or synthesis articles. Statistical methods (meta-analysis) may or may not be used to analyze and summarize the results of the included studies.  Reports of systematic reviews and meta-analyses must include a completed [PRISMA (Preferred Reporting Items for Systematic Reviews and Meta-Analyses)](http://prisma-statement.org/) checklist and flow diagram to accompany the main text……………………….  Authors must also state in their “Methods” section whether a protocol exists for their systematic review, and if so, provide a copy of the protocol as supporting information and provide the registry number in the abstract.’  According to this guidance the review we report in our manuscript does fulfil the criteria of a systematic review. It had a pre-specified research question (how does the communication of risk information in clinical trials influence decisions to participate and what are the best methods to improve understanding in a trial context?) that used explicit systematic methods to identify and select relevant research (methods outlined on pages 6 and 7), and to collect and analyse data (methods outlined on pages 7 and 8). We did not use statistical methods to analyse and summarise the results as this would not have been appropriate given the heterogeneity of the included studies – this is also deemed acceptable by PLOS ONE guidance for systematic reviews. We included a completed PRISMA checklist and flow diagram to describe and provide transparency to the methods. We also provided an electronic link to the review protocol which is publicly available.  The reviewer makes the point that ‘the papers are themselves so varied that no conclusions can be drawn except at the individual study level’. This does not mean the review is not a systematic review – this is a limitation of the included studies, not the process. | |
| **6** | The main problem is in the definition of the problem to be studied: The authors restrict themselves to papers about clinical trial contexts. The logic for doing so is based on the idea that risk communication in a clinical trial context is somehow fundamentally different than other contexts. It therefore excludes many other, very similarly structured studies examining risk communication in other kinds of decisions. While systematic reviews are very valuable way of considering a range of papers that have studied similar questions, the current set of papers are not really "similar" in important ways. Thus, the criteria is both narrow (in focusing on clinical trials situations only) and very broad (in allowing in all kinds of risk communication of very different types).  **Response**  As Reviewer 2 highlights in their second comment, ‘participation in a randomized clinical trial actually has a degree of complexity, uncertainty, risk much larger than choosing a treatment.’ We have tried to outline the differences between decisions about treatment and decision about trial participant and the influence understanding of risk may have on these in the Introduction on page 5. We have now revised this section to provide further rationale for why risk communication and its influence on decision making may be different in a trial context compared to treatment. Lines 81-89.  These (and other) differences in decision making was the reasoning for excluding studies examining risk communication in other types of decisions e.g. for treatment, where although there may be methods for best practice (which we point to in the discussion section) these have not been shown to be best practice for trial participation decision and should not be assumed to be directly transferable without testing. Also see response to point #7 below.  As mentioned before, although these studies are not ‘‘similar’ in important ways’ – this is a limitation of the field – not of the process of the review. | |
| **7** | I can't really complain about the analysis because the analysis is entirely individual paper focused. Each study is summarized, but there is no real integrative analysis. Because of its narrowness, little of the risk communication lessons derived from other work are discussed. This leaves readers with the mistaken impression that little is known about how to effectively communicate the types of risk information relevant to clinical trials. What is lacking (as this paper shows) is empirical studies showing that these lessons carry over to this context. The unfortunate reality is that the lack of relevant studies is because there is little incentive for researchers to do such conceptual replication studies when the answers seem so obvious.  **Response**  We have tried to integrate learning from other risk communication studies within the discussion section to highlight examples of good practice from other fields/areas (lines 398-411, 421-425, 477-456 ). However, given the differences between decisions about trial participation treatment these methods will require formal evaluation in a clinical trial setting before effectiveness can be assumed (See statement on Lines 506-507 regarding need for future research).  In addition , we have now included a paragraph to highlight examples of best practice that should and could be looked to to inform many of these future studies. Using empirical findings from a treatment context identified as best practice to determine whether these are transferable to a trial participation context – lines 501-507. | |
| **8** | Given the limitations of the data, i am also concerned by the length of the discussion section and the degree that it tries to draw conclusions from single studies (which is antithetical to the concept of a systematic review).  **Response**  We have edited the discussion as stated in response #7 above.  In addition, we have attended to the reviewers concerns regarding conclusions based on single studies by removing any recommendations and reverting to highlight the lack of an existing evidence base and the need for further research. | |
| Reviewer #2 | | |
| **9** | | The paper reports a systematic review of studies addressing the effects of various methods to communicate the patients about the risks of participation in RCTs of treatments. They found 7 studies after considering almost 5000 titles. Of the 7, five were RCTs (of communicating about RCTs) and two had a different design (prospective, non randomized). Additionally, NONE of these studies were done with patients who actually considered participation in a clinical trial for their own disease. Rather they asked the participants to hypothetically pretend they had a disease and decide whether they would participate in a trial of a fictional treatment for the disease. This is a limitation of the field, not of the review. Because the studies did not use the same interventions nor measure the same outcomes, it was not possible to summarize the effects of the communications quantitatively (meta analysis), so they did a narrative summary. What this means in practice is that they summarized in extensive detail the methods and findings of each study, in alphabetical order of authors, grouped by whether the study was an RCT or not. My perception of the paper has two high level comments.  **Response**  No response required |
| **10** | | Comment 1. The descriptions of the studies have too many details, described “telegraphically”. That is, you almost have to go read the paper to understand what was done. An example is this extract from the description of the second study described: “three different risk/benefit formats: text (e.g. risks and benefits described as number out of 100 experiencing outcome); , tables (e.g. same as text based but presented in tabular format); or pictographs (e.g. a matrix of 100 rectangular blocks representing 100 children then coloured to represent presence (blue) or absence (grey) of outcome). The presence or absence of a risk severity graphic was also investigated. This graphic was described as having increasing risk represented by blocks of different sizes and colours (yellow through red), and an arrow depicting where the risk or side effect fell on the scale accompanied by a description of risk as ‘mild’, ‘moderate, or ‘severe’. Risks and benefits of two drugs for post-operative pain in children were communicated in one of three formats and presented as either absolute risk or incremental risk to participants.”  While I am sure these statements accurately describe the elements of the study, it is difficult for the reader of this summary to get a sense of the design – was it 3 by 2 by 3 by 2, i.e., 36 different cells? Or 12?  **Response**  We have tried to revise the description of included studies such that they provide enough detail to describe what was done but succinctly enough such that the key information is conveyed. Several sections throughout.  On going back through the description of studies to address this comment we identified a mistake in the included data of one of the studies. The data included from the Tait *et al* study [ref 23] was extracted from the wrong paper. The reference cited in the reference section is correct (Tait AR, Zikmund-Fisher B, Fagerlin A, Vopel-Lewis T. Effect of various risk/benefit trade-offs on parents’ understanding of a paediatric research study. Paediatrics. 2010. 125(6). e1475.) but the extracted data came from a linked study (Alan R. Tait, Terri Voepel-Lewis, Brian J. Zikmund-Fisher & Angela Fagerlin (2010) The Effect of Format on Parents' Understanding of the Risks and Benefits of Clinical Research: A Comparison Between Text, Tables, and Graphics, Journal of Health Communication, 15:5, 487-501, DOI: [10.1080/10810730.2010.492560](https://doi.org/10.1080/10810730.2010.492560)) but is not risk communication in a clinical trial. The wrong paper had been stored electronically. This error has now been corrected and the correct data included and edits made accordingly. We have also now re-checked all data from included studies to ensure no further mistakes. Lines 308-323 |
| **11** | | Comment 2. The introduction presents “participation in an RCT” as just another area in which physicians might need to communicate about risks to patients, as if it were a routine drug or an operation. The reader might ask, what is new here, it is just a different domain in which risks must be communicated. Surely we’d expect that communication aids or approaches that work for other treatment decisions would similarly work here. But participation in a randomized clinical trial actually has a degree of complexity, uncertainty, risk much larger than choosing a treatment. While the tradeoffs between alternative treatments involve risks and value tradeoffs, the tradeoffs between a study or not doing a study involves a) a loss of control, a LACK of being informed, and b) greater uncertainty about the benefits. I say greater uncertainty, because while treatment A has its own uncertainties (will it work? Will there be side effects?), a trial in which treatment A is one of the options to which one will be assigned has all those uncertainties, plus the uncertainties about the alternative (experimental) treatment whose uncertainties are even greater, plus the uncertainty about which of the two (or more) arms one will be assigned to. Quite possibly the communication aids or approaches that work for a decision among accepted treatments with (somewhat) known success probabilities would NOT work for this more complex situation. It would be helpful for motivating the reader to engage with the paper if this difference between communicating the risks of RCTs, versus simple (?) treatments, were stated explicitly. It makes the paper more interesting. It would also be interesting if, in describing the studies, the review noted whether and how the papers acknowledged and addressed the added complexity of the participation decision. Did they communicate ONLY about the probabilities of treatment assignment? Or did they name the good and bad outcomes that could occur with either treatment? Did they explicitly state that the uncertainty about the benefits and side effects of the experimental treatment is greater than the uncertainty about the benefits and side effects of the currently accepted, comparison treatment? That is, what in particular was the “risk communication” about?  **Response**  We agree with the reviewer that decisions about trial participation are more complex than decisions about treatment and the weighing up and trade offs of risk and benefit are likely more involved. We have included additional text on lines 81-89 to reflect this.  With regard to the latter point relating to whether probabilities were presented of treatment assigned versus both treatments, it is assumed that as these studies were hypothetical, patients were not allocated to an intervention. We were able to identify whether the interventions being evaluated included risk communication relating to the intervention or comparator. We have now included that information in Table 3 under ‘content’ of intervention. We have also further commented on this within the discussion section lines 458-463. |
| **12** | | The paper is somewhat verbose; partly in the attempt to be very clear about what is being said, but it could be tightened.  **Response**  We have tried throughout the manuscript to tighten up the language to make the arguments clearer and the writing more succinct. |
| **13** | | Particular suggestions. (Some of these suggestions may be too minor, or wrong. But the reviewer instructions say that there is not going to be any copy editing on the part of the journal. Your $1500 is not sufficient to buy that service.)  Abstract, line 19. “context” should be “contexts”.  **Response**  Edited accordingly |
| **14** | | Line 27. When first encountered, it is difficult to understand what “studies set within hypothetical host trials” refers to. I have interpreted it as “studies in which risk information is communicated to support the participant’s hypothetical decision to participate in a fictional trial of a fictional treatment for a disease that the participant does not have”, but that is not what the words “set within a hypothetical host trial” means.  **Response**  We have now edited this sentence to make it clearer to the reader what ‘studies set within hypothetical trials’ actually are. Lines 187-191 |
| **15** | | Line 31. “In term so” should be “in terms of”.  **Response**  Edited accordingly |
| **16** | | P 4, line 50. Expand “participants approached to take part and those consented to trials” to ““participants approached to take part and those who consented to participate in trials”  **Response**  Edited accordingly |
| **17** | | Line 51, participants.  **Response**  Edited accordingly |
| **18** | | Line 57, participant should be plural.  **Response**  Edited accordingly |
| **19** | | Line 66, 67, change “risk communication with regard to public health messages, health and treatment decisions for patients, and in screening contexts” to “risk communication with regard to public health messages, health [behavior?], and treatment and screening decisions for patients”. I guess “screening” may be done on people who are not technically “patients”, but so often it is done in primary care clinics.  **Response**  Edited accordingly |
| **20** | | Line 88, change “findings from work from our group has shown” to “findings from our group have shown”?  **Response**  Edited accordingly |
| **21** | | Line 91, change “highlighted patients preferences” to “highlighted that patients’ preferences”.  **Response**  Edited accordingly |
| **22** | | Lines 93 and 94, move “from treatment and screening decisions” to after “decision making” in the next line, and change “from” to “about”.  **Response**  Edited accordingly |
| **23** | | Last sentence before “Methods”. Participants should be possessive.  **Response**  Edited accordingly |
| **24** | | Methods. I am not sure what an “explanatory” study is. I don’t think it is an “exploratory” study (though that is used in next paragraph, but those studies were excluded). Does it mean, “a study of ways of explaining something to a patient”? If that is what it means, I don’t think the phrase “explanatory study,” without more elaboration, will be understood.  Style. “were included” was used three times in this one paragraph.  **Response**  We have edited the first paragraph of the methods section to clarify what is meant by explanatory. We have also removed two of the ‘were included’ statements. studies (See lines 124-130).  We have also amended the exclusion criteria to clarify what is meant by exploratory studies – see lines 135-139. |
| **25** | | Lines 199 and 120, I don’t think it needs both “exploratory studies” and “that have explored”. Maybe drop the first.  **Response**  See response to point #24 above. |
| **26** | | Lines 124 and 125. What is a “senior information officer”? a modern librarian?  **Response**  An Information Officer is often a term used to describe librarians who carry out library/information functions but not in a library. We have chosen to retain this term within the text. |
| **27** | | Line 129. Say May 10th is in 2019.  **Response**  Edited accordingly |
| **28** | | Line 138. “and progressed for data extraction procedures”? does than mean, “were followed by,” “led to”, “progressed to”?  **Response**  Edited accordingly |
| **29** | | Line 142. Given the immediately preceeding sentence, change “The seven studies deemed eligible and identified for data extraction were summarized” to “The seven selected studies were summarized”.  **Response**  Edited accordingly |
| **30** | | 146. I’m not sure the Journal’s conventions. I would add commas around “i.e.” and “e.g.”, through out, “by content of intervention, i.e., probability” not “by content of intervention i.e. probability”  **Response**  Edited throughout to include a comma. |
| **31** | | Data extraction and management. Line 151. Data were, not data was.  **Response**  Edited accordingly |
| **32** | | Lines 151 and 152. Change “summary data was extracted regarding the host trial for each study and summarised in table form” to “summary features of the host trial for each study were summarised in table form”. Of course you had to extract it before summarizing it, but it need not be said.  **Response**  Edited accordingly |
| **33** | | Line 155. Space after the comma.  **Response**  Edited accordingly |
| **34** | | 4 instances of i.e. without surrounding commas in this one paragraph.  **Response**  Edited accordingly to include surrounding commas around the i.e. |
| **35** | | Line 160. Change “satisfaction with communication method; and level of decisional conflict” to “satisfaction with communication method, and level of decisional conflict”  **Response**  Edited accordingly |
| **36** | | Lines 168 169. Here might be a place to give an example of how a study of communication about the risks of RCTs might be “set within a hypothetical host RCT”.  **Response**  We have now provided an example of a scenario from one of the included studies that outlies how participants are presented with information and asked to consider participation in a hypothetical clinical trial. Lines 187-191 |
| **37** | | Line 170, drop unnecessary words, in the context. Change “The seven included studies that explored interventions to explore risk communication during informed consent varied by design” to “The seven included studies had various designs”.  **Response**  Edited accordingly |
| **38** | | Line 172. Could “prospectively designed” be simply “prospective”?  **Response**  Edited accordingly |
| **39** | | P 9, line 179. “an” not “and”  **Response**  Edited accordingly |
| **40** | | Line 180, “included” is ambiguous. Does it mean one study was only of parents, or one study had not only patients deciding for themselves but it also included some parents deciding for children?  **Response**  We have now edited this sentence to make it clearer that this study included only parents who were considering participation for their child. |
| **41** | | Lines 186-188. It was unclear to me at first that these were the authors of the studies you were summarizing; at first I thought they were references for the kinds of method used, e.g., probability presentation, risk framing, risk interpretation. Be more clear.  **Response**  Rather than including the authors first name for these citations we have reverted to the numbered system so as not to cause any confusion for the reader. Lines 209-212. |
| **42** | | 194. Hypothetical is unclear. Is it that the participant is hypothetically making a decision about a real study, or is hypothetically making a decision about participation in a hypothetical or fictional study? The word is too vague for the many possibilities.  **Response**  Given we have already highlighted earlier in the manuscript that all of the included studies included those set within hypothetical RCTs, we have chosen to remove ‘hypothetical’ from this sentence to avoid confusion. |
| **43** | | 194. But since you’ve already told us all the studies involve pretending to decide about a fictional study, if I interpret correctly, you do not need to repeat it every time you describe a study. It just confuses the reader.  **Response**  Please see response to #42 above. |
| **44** | | Line 197. Is the reference a cite of an included study, or of the paper describing numerical values associated with words like “common” in an official EU document? Or of an earlier study of the EU prescribed communication language?  **Response**  Reference 22 cited here is an included study – Berry & Hochhauser 2006. |
| **45** | | Line 199. Add “were” before “asked”.  **Response**  Edited accordingly |
| **46** | | P 10, lines 208 and 209. Put quotes around the terms common, uncommon, and rare.  **Response**  Edited accordingly |
| **47** | | Line 217. Drop “t”.  **Response**  Edited accordingly |
| **48** | | Line 219, and extra comma.  **Response**  Edited accordingly |
| **49** | | Line 225. 2 by three by 2 design? Or did each get info about both drugs in the same format?  **Response**  See response to #10 above |
| **50** | | Line 230. Move “(66.55) back to after “gist understanding”.  **Response**  Edited accordingly |
| **51** | | 234. orphan “).”  **Response**  Edited accordingly |
| **52** | | 239. Comma after “respectively)”?  **Response**  Edited accordingly |
| **53** | | 239. Comma after graphics.  **Response**  Edited accordingly |
| **54** | | 252. space after 8.3. comma after “scale”.  **Response**  Section now deleted to address other comments |
| **55** | | 259 to 262. The instructions in this study were, ‘Out of 100 people whose lives would likely be cut short by heart disease and  260 begin taking this drug, we expect that 95 will show substantial improvements in their chance of 261 survival and 5 will show no improvement in survival’. Do you want to comment on how vague and uninformative that is?  **Response**  We have now included a sentence within the discussion to highlight that the lack of detail contained within these statements brings into question participants understand of the nature of the risks/benefits. Lines 443-445. |
| **56** | | 268 to 271. “The majority of participants (59%) chose to take part in the trial when outcomes were framed as losses, however a greater majority of the ‘gain’ group (65%) declined to participate. When both framings were presented, 62% of participants chose to participate, making a similar choice to the ‘loss’ group.” I find the switch in reference to be confusing.  Who cares the relative size of the majority? Keep it consistent: “The majority of participants (59%) chose to take part in the trial when outcomes were framed as losses, while only 35% of the ‘gain’ group chose to participate. When both framings were presented, 62% of participants chose to participate, making a similar choice to the ‘loss’ group.”  **Response**  Edited accordingly |
| **57** | | 278 needs a period at the end.  **Response**  Edited accordingly |
| **58** | | 313 to 317. This discusses inaccuracy of patient mapping of numbers onto EU definitions of probability words. To interpret this, one would need to know what all the options are. Are they missing by one category, or are they several off? But the larger question is, what is the relevance of this to communicating about the risks of participating in randomized clinical trials? This seems a general problem with any risk communication. Of course, it is part of the whole comprehension process.  **Response**  We have deleted lines 360-362 as we agree with the reviewer that this data relates to a more generic point about risk communication and doesn’t add anything to the considerations for trial participation decisions. |
| **59** | | Line 326. Change “for only benefit” to “only for benefit”?  **Response**  Edited accordingly |
| **60** | | Line 326. I’m not sure what “saw” means. Was this a study intervention, or a participant behavior, picking up on an implication that is present? I think it is the former. I think it is a situation of: show one of three formats; see how many consented. If that is what it is, then you are describing it backwards, in terms of causality. “of those who consented, X saw a format” is the reverse order. The interesting question is, “of those who were shown x format, who saw x format, what proportion consented? I think this needs to be restated.  **Response**  This section has now been edited to provide a clearer description of the study and to clarify what ‘saw’ means in this context. Lines 371-374. |
| **61** | | Line 327. Not clear what it was “to see neither”  **Response**  See response to #60 above. |
| **62** | | Line 339. “aspect” should be plural.  **Response**  Edited accordingly |
| **63** | | 345. “identified” seems an unnecessary word.  **Response**  Edited accordingly |
| **64** | | 354. “deemed”. Is this that the participants reported the pictographs as more helpful, or that the authors of the reviewed studies concluded based on their data that the pictographs were more helpful. Who is “deeming”.  **Response**  Section now deleted to address other comments. |
| **65** | | 355. Not clear what the distinction is between a pictograph and a severity graphic, why is the one good and the other bad?  **Response**  Section now deleted to address other comments |
| **66** | | In this discussion, I feel a need for the authors to more authoritatively criticize the reviewed studies, not just to summarize them. I wonder if it would be helpful to have a model of comprehension of risk communication, e.g., the required steps: a) comprehend the scale, b) use the info to interpret one’ situation and guide action. Of course, if they fail the first step the second step has little chance of happening – unless they get it from alternative clues like verbal summary of gist. Perhaps you get at that in line 371.  **Response**  Linked to various other comments we have tried to include a more critical stance of the included papers and link back to basing new studies on existing standard from risk communication in a treatment decision making context. Edits throughout. |
| **67** | | Line 363. Change “and verbal conditions, however it did find” to “and verbal conditions; however, it did find”  **Response**  Edited accordingly |
| **68** | | Line 371. Change “as well as comprehending the concept” to “nor to comprehend the concept”. Preserves parallel with the previous clause.  **Response**  Edited accordingly |
| **69** | | 373 to 374. It would help if the manuscript more clearly separated the paragraphs. This is an example where it is hard to tell.  **Response**  Edited accordingly here and throughout |
| **70** | | Line 374. Put “The” before “Sutherland”.  **Response**  Edited accordingly |
| **71** | | Line 379. “generally”? should that be “usually”? Mathematically, “generally” means always true.  **Response**  Edited accordingly |
| **72** | | 382. I think it is “their best interest” rather than plural, but I’ve seen it both ways.  **Response**  Edited accordingly |
| **73** | | 390. “when gains were framed as gains”? “when outcomes were framed as gains”.  **Response**  Edited accordingly |
| **74** | | 403. sheets = leaflets. If you don’t say that, the reader does not know what “PIL” means.  **Response**  Edited accordingly |
| **75** | | Lines 406 407. This list is hard to interpret, the elements do not seem parallel, and it is hard to tell if “understanding of” applies only to “trial processes”, or to all three elements in the list.  **Response**  Edited accordingly |
| **76** | | 416. Not sure the meaning of “its evidenced importance”. It’s evident importance (on the face of it), or its proven importance (proven by lots of previous studies, including the evidence reported by study 17)?  **Response**  Edited accordingly |
| **77** | | Line 426. I’m not sure if agreement should be “number… means” or “number of studies… mean”.  **Response**  Edited to ‘means’ |
| **78** | | 428.”hypothetical nature of the studies”. But these were real studies, actually done. Need a different way to talk about this. And it is ambiguous if you mean “the 7 studies we reviewed” or “the stimulus studies, presented to the participants in the studies”.  **Response**  We have now edited this section to provide further information to expand on the explanation of hypothetical. Line 490-493 |
| **79** | | Line 432. I don’t think “however” obeys the same rules as “but”. So I think the punctuation should be not “earlier phases of clinical trials, however only one of the studies” but “earlier phases of clinical trials; however, only one of the studies”.  **Response**  Edited accordingly |
| **80** | | Line 433. I think however needs to be set off on both sides by commas.  **Response**  Edited accordingly |
| **81** | | Line 440. What does “it” refer to?  **Response**  Here ‘it’ refers to understanding and as such we have replaced it to reflect that fact. |
| **82** | | 443. “most useful” compared to what, or out of what set of options for this particular situation? Or do you mean more useful in this situation than in other situations where it might be used?  **Response**  Section now deleted to address other comments |
| **83** | | 444. I think it is “complementary” not “complimentary”.  **Response**  Edited accordingly |
| **84** | | In the reference list, words are capitalized inconsistently. Some titles have all the words capitalized (appropriate for book titles but not paper titles) while others have just the first word capitalized. Be consistent, and consistent with the journal standards.  **Response**  Edited accordingly |
| **85** | | Reference 25 needs a period between the title and the journal name.  **Response**  Edited accordingly |
| **86** | | Reference 38. Laura siminoff’s paper had only one page?  **Response**  Edited accordingly |
| **87** | | Figure 1. I note that you found 3 systematic reviews – how did they differ from the present systematic review? Should they be included in your literature review (not just in your characterization of your systematic review’s rejects).  **Response**  The systematic reviews identified in the literature search were different from the review detailed in our manuscript as they either focused on the ‘best’ methods for informed consent for trials but without considering risk communication, or, they reviewed the literature on communication of risk but in a treatment context. Given these reviews were not within scope or deemed eligible for inclusion they were not included in the review. As the reason for exclusion was their scope/content rather than the fact they were systematic reviews, we have amended Figure 1 to make this clearer and removed the reference to Systematic reviews within the ineligible studies box and subsumed the 3 papers within the relevant categories (1 into ‘Not focused on risk communication’ and the other 2 into ‘Not about clinical trial participation’). |
| **88** | | Table 2. Why a date for Tait but not the others.  **Response**  Edited accordingly from Table 2 and from Table 1 |
