# Supplementary material for: A systematic review of risk communication in clinical trials: How does it influence decisions to participate and what are the best methods to improve understanding in a trial context?
Source: PLoS One. 2020 Nov 16;15(11):e0242239. doi: 10.1371/journal.pone.0242239 (PMC7668608; doi:10.1371/journal.pone.0242239)
Supplement: Attachment — Submitted filename: PONED1919791_Response to reviewers_140920.docx [file pone.0242239.s004.docx]

| **Title** | **A systematic review of risk communication in clinical trials: how does it influence decisions to participate and what are the best methods to improve understanding in a trial context?** |
| --- | --- |
| **Authors** | Maeve Coyle and Katie Gillies |
| **Manuscript ID** | PONE-D-19-19791R1 |
|  |  |

Dear Editor,

Thank you very much for considering our manuscript for publication in PLOS ONE.

As requested please find below a list addressing the second set of queries raised by the reviewers. We again thank the reviewers for their considered responses and we believe that in addressing these we have significantly strengthened the manuscript.

I look forward to hearing from you.

Best wishes

Katie Gillies (on behalf of the authors)

| **Editors comment** | |
| --- | --- |
| Generally the reviewers are happy with the review. Much of the problem focuses on the nature of the review. PLOS ONE accepts systematic reviews but not narrative reviews. However, reviewer #1 raises an important point that the pure systematic review that concludes that there isn't enough evidence is useful but not very helpful. On the other hand a more narrative review might be more helpful. I think that there may be a compromise here. Maintain the systematic review but have a more detailed analysis of the topic in the discussion. Thus, it is a systematic review with a  more comprehensive analysis.  **Response**  We have now updated the paragraph on risk communication in treatment and/or screening to further extend the provision of the evidence available on risk communication in this context. We haven’t provided an extensive discussion of the evidence in this space as there are several systematic reviews on this topic which do that. Most notably an overview of reviews conducted by NICE in 2014 and a further review published after this overview that focused on risk communication. We weren’t previously aware of these reviews and both highlight that the quantity and quality of evidence in this area is limited. As such we can’t make strong recommendations on what learning can be directly applied but do highlight the key findings of each on the discussion. We have, however, also included discussion of several good practice recommendations which have been generated from empirical research and are underpinned by evidence (albeit sometime low quality). We hope that the Editor and Reviewer now feel that this extension of the discussion goes some way to propose how existing guidance could be applied to this context.  See page 19 line 439-488. | |
|  | |
| **Reviewer #1:** | |
| **1** | This revised manuscript reports a systematic review of studies examining different methods of presenting risk and benefit information in the context of clinical trial consents. The authors required the studies to be examining communication in a clinical trial context, resulting in only 7 widely varying papers which are then summarized one at a time. The primary conclusion is that there is little extant guidance for presenting risk and benefit information to guide clinical trial participation decisions.  In my previous review, I expressed significant concerns about the authors' framing of the scope of this review. In particular, the restriction to studies whose scenarios / tasks could only be about clinical trial decisions results in a small, heterogeneous set of papers and does not consider the relevance of structurally similar papers addressing other contexts such as treatment or screening decisions. The resulting manuscript thus is more descriptive than integrative, and it conveys a primary message of "we don't know", which is literally true but practically false (we do know a lot that has been demonstrated to generalize across decision contexts).  In their response to my review, the authors reiterated that their work aligns to the process of a systematic review. Such was not actually my concern, and I agree that their work does in fact follow that process. My concern is that, in this context, a true systematic review is less valuable to the field and less useful to practitioners than a review that is more integrative.  The issue is actually simple: I fully agree that actual clinical trial participation decisions are different in some important ways from actual treatment decisions. I am quite skeptical that _hypothetical_ clinical trial decisions (of the type studied in the reviewed papers here) are actually that different from _hypothetical_ treatment decisions because many of the differences in real-world mindsets are gone, replaced by the common experience of reading hypothetical scenarios. More importantly, I fundamentally disagree with the idea that the process of _communicating_ _risks_ is so categorically different in clinical trial contexts that the exclusion of all other studies, and the resulting conclusion that little is known about how do risk communication well in this context, is justified. The authors do not provide a clear argument how the comprehension task, the interpretation task, and/or the integration tasks associated with receiving and using risk information in this context are different from those same tasks in, say, a decision support tool for treatment or screening decision making.  **Response**  As per the Editor’s suggestion we have now updated an extended the discussion section on risk communication in treatment and screening to identify opportunities for learning across contexts. See page 19 lines 439-488. |
| **2** | The authors have made several relatively minor revisions that acknowledge that "lessons from effective risk communication in a treatment and/or screening context can provide examples of best practice that could be used for those developing PILs for patients considering clinical trial participation" and that future research could use "the best practice examples from treatment decision making as a starter" They also now refer to one of a number of relevant risk communication reviews to begin orienting readers to that literature. I thank the authors for these steps, as they do mitigate my concerns somewhat. But, I am not sure that they are enough to offset the primary conclusions (as still stated in the abstract) that "there is as yet no clear optimal method for improving participant understanding," despite lots of evidence from other non-included studies that certain approaches are likely to be more effective than others.  **Response**  We recognise the reviewers concerns and are pleased that they believe we have addressed these in part.  The purpose of this review is two fold: 1 to summarise the evidence that already exists in this space, but also importantly 2.to identify that a gap in the empirical knowledge base does exist and that we cant assume the transfer of learning from treatment and screening will be directly relevant to decisions about trial participation. We have provided evidence from empirical studies in the Introduction section (pages 5 and 6) that highlight that the perception risk in clinical trials has particular contextual nuances which seem to influence perception of risk and therefore it cannot be assumed a direct translation from previously literature demonstrating best practice will also hold here.  However we take on board the reviewers concerns that for the initiated it may come across an absence of evidence across fields and this is not correct. Therefore, alongside the edits to the Discussion section we have tried to further address the remaining concerns by editing the study conclusions both in the abstract and in the conclusion section to point back to treatment and screening literature. See page 3 lines 41-43 and page 22 line 516. |
| **3** | My preference would be for an option that I suspect the authors (and perhaps the journal) would reject: To create a kind of hybrid paper, one that does the systematic identification of papers and notes the dearth of relevant empirical evidence (which, to me, is the primary value of this paper) but then complements it with a much more detailed and somewhat speculative discussion of _specific_ principles of good risk communication evident in the broader literature that are relevant in clinical trial contexts. This type of paper could be quite valuable to many audiences in ways that I fear that the current manuscript will not be.  **Response**  See responses to previous comments |
| **4** | In net, I don't have much in terms of small comments. The authors have been generally responsive to the detailed comments provided by other reviewers, and they have taken some steps towards addressing my own comments. I just remain quite concerned that the takeaway message of this paper is not particularly helpful (and may be harmful) in guiding improvements in risk communications in clinical trial documents.  **Response**  See responses to previous comments |
| **Reviewer #2** | |
| **5** | I read the review with better comprehension this time, thanks for working to organize and clarify it. Thank you |
| **6** | There are some verbal expressions that confuse me. It may be that the UK and the US are “two countries divided by a common language”. One is the characterization of the studies as having been “nested in” or “hosted in” or even “within” a hypothetical trial. From reading the paper the situation is just that the studies were done with a cover story or situation (a trial for a disease that the participant does not actually have) and the information about the situation is varied. The “hosting” and “nesting” and “within” convey that there is something bigger going on, say a study with multiple interviews over time, and that an incidental add on is that on one occasion among the 4 questionnaires filled out that day is the booklet that contains the study of risk communication. But I don’t find any evidence for this. Is this part of the way people in the UK talk is it an idiosyncratic choice of words just this once? If the latter, it is confusing for this reader and it seems to add unnecessary work to the task of reading the review. There was some complaint about this last round, and the promise in the author’s response that it had been fixed, but it does not seem to have been addressed.  **Response**  Within trials methodology research there has been challenges with how to describe studies set within clinical trials. Recently these have been given the acronym SWATs (Studies Within A Trial) and there is now published guidance on what SWATs are ^1^, when further evaluation is required^2^, and how to report these studies (in preparation). Much of the discussion ahs arisen in relation to methodological trials (e.g. evaluating different risk communication strategies) set within clinical trials (e.g. evaluating a healthcare intervention) and how you can appropriately communicate the distinction between the two in a publication. Hence there have been suggestions to refer to the clinical trial as the host or parent trial and then consider situation the methodological trial by using descriptors such as nested or embedded within the host trial. We had used similar language to recent trials methodology review^3^ which also talks about host trials.  It may be that this use of language in this way is a difference between the UK and the US but then this likely exists for other countries too. Given PLOS One has an international readership we have removed the reference to ‘host’ or ‘nested’ as much as possible throughout the manuscript. However, there is one section where we felt it was important to use this term to provide the distinction between the host clinical trial and the methodological trial. We have now included a description of what we mean by host trial to avoid confusion. Please see edits on Page 8 line 166 We have chosen not to exclude the use of the word ‘within’ when referring to the studies being set *within* a trial as we believe this provides further context important for the reader to be reminded of.  ^1^Treweek, S., Bevan, S., Bower, P. *et al.* Trial Forge Guidance 1: what is a Study Within A Trial (SWAT)?. *Trials* **19,** 139 (2018).  ^2^Treweek, S., Bevan, S., Bower, P. *et al.* Trial Forge Guidance 2: how to decide if a further Study Within A Trial (SWAT) is needed. *Trials* **21,** 33 (2020). <https://doi.org/10.1186/s13063-019-3980-5>  ^3^Rowlands C, Rooshenas L, Fairhurst K*, et al*  Detailed systematic analysis of recruitment strategies in randomised controlled trials in patients with an unscheduled admission to hospital  *BMJ Open* 2018;**8:**e018581. doi: 10.1136/bmjopen-2017-018581 |
| **7** | On the presentation of the reviewed studies’ findings, in the Results section. I note that when comparisons are made, the statistic presented is the p value. Only a few times was there an effect presented (X% versis Y%). Quite possibly those numbers were present in the previous version and have been removed because of the complaint of “too much unreadable information”. So I need to be careful here. I know that the social science statisticians, such as the American Psychological Society (to which the authors need not have any loyalty), ask for an effect, a degree of freedom or N, as well as the p value. And then there are the new standards of presenting the effect estimate and confidence intervals. But these are for primary studies. For reviews, however, the same general ideas are still relevant for the reader to understand how much weight to put on a study. Degrees of freedom. While the text need not have a df next to every p, how about when the study is first described, saying how many people were in it, in each group? Then we can know if it was 10 or 1000, and form opinions accordingly.  **Response** As per the reviewer’s suggestion we have included the total number of participants in each study and , where appropriate, indicate the number of participants in different groups. See changes tracked in Result section. |
| **8** | Effect size. Of course there is the issue of statistical significance compared with clinical or practical significance. If we knew the N was huge and the effect was p = .05, we might suspect the effect is actually small and of little practical importance (in the absence of the text giving the effect size). But really, for at least half of the “(p < .xxx)” statements, providing a simple idea of what the compared means were might be a great help. If probabilities, we know what those scales mean. Sometimes if it really would help understand the effect, you might need to explain the scale.  **Response**  We have added back information from the previous version of the review on measurement scales for each study. See tracked changes in Results section. |
| **9** | Abstract. “participants” needs to be plural possessive in this: “studies that aimed to support potential trial participants decision”.  **Response**  Edited accordingly |
| **10** | P 4. First para, I suggest changing “should include explanations of the ‘reasonably foreseeable risks or inconveniences’ and expected benefits, and where there are no clinical benefits to the participants, they must be made aware of this” to “should include explanations of the ‘reasonably foreseeable risks or inconveniences’, expected benefits, and where there are no clinical benefits to the participants, they must be made aware of this”  **Response**  Suggested edit is already represented in text. |
| **11** | P 5, line 78. “Decision” should be plural.  **Response**  Edited accordingly  I suggest changing “Conditional altruism is the concept that participation in the trial will benefit society but there is a need for a benefit (which is influenced by perception of risk) for self to be tied in” to “Conditional altruism is the concept that participation in the trial will benefit society but there must be a benefit (which is influenced by perception of risk) for self”.  **Response**  Edited accordingly  Suggestion: change clumsy punctuation from “trade-offs between risk and benefit in a trial involve; layers of complexity in addition to those for treatment such as: loss of control over which treatment they receive; and potentially greater uncertainties as often participants have to consider the risks and benefits of a minimum of two competing treatments” to “trade-offs between risk and benefit in a trial involve layers of complexity in addition to those for treatment, such as loss of control over which treatment they receive and potentially greater uncertainties, as often participants have to consider the risks and benefits of a minimum of two competing treatments”  **Response**  Edited accordingly |
| **13** | P 6, line 101. Add “that” after “highlighted”.  **Response**  Edited accordingly |
| **14** | Line 121 “within information presented” These words seem redundant. Can they simply be deleted?  **Response**  Edited accordingly |
| **15** | P7, line 126. Add “studies of” after “include”.  **Response**  Edited accordingly |
| **16** | Lines 130-131. What does this mean? I don’t understand what kinds of studies were excluded. “More eStudies investigating participants perceptions of receiving risk communication…”  **Response**  Edited accordingly – this was an error and has now been edited. |
| **17** | Is an “eStudy” a thing? Why exclude it?  **Response**  As above |
| **18** | Also, “participants” needs to be plural possessive.  **Response**  Edited accordingly |
| **19** | I understand that you consider a “Senior Information Officer” to be distinct from a “librarian”, as stated in response letter. But simply leaving it as such does not solve the problem that for people in different university systems, there is no such thing. Could you explain it in a phrase? Is it like a police officer, the “fact police”. That would be interesting. In my area, “public information officers” are public relations flacks, and then there are “information systems security officers”, but I would not go to either one for help with a library search.  **Response**  We have now amended the title of this role and add a brief descriptor in parentheses which hopefully enables further understanding for the reader. See Page 7 lines 139-140. |
| **20** | P 9. Line 183. “…the studies asked participants to imagine they were being recruited into a host clinical trial, provided brief information about the host trial (such as clinical population, intervention, comparator, outcomes, etc), then provided various formats of risk communication (such as verbal or numerical descriptors) and assessed relevant outcomes.” “assessed relevant outcomes” is ambiguous. Is this part of the participant’s task (to assess relevant outcomes that are described), or is “assessed” an adjective describing a part of the materials that the participants are shown; where someone else has assessed the outcomes (probably verbally, versus numerically) and the participant looks at it and does some other judgment (e.g, whether to sign up for the trial). I think it is the latter. If so, changing “assessed” to “assessments of” would make it clearer.  **Response**  Edited accordingly |
| **21** | Line 186. An example of my problem with “nested in”. How about “in the context of” or even just “about”?  **Response**  Edited accordingly – please see response to point 6 above. |
| **22** | In 190, again, “set within a trial” just means that the context of the vignette was a non drug intervention rather than a drug.  **Response**  Please see response to point 6 above. |
| **23** | P 10. Line 198, explanation of structure of upcoming review. Again, “embedded” is a confusing word that could simply be dropped! I did not know whether the two groupings --first design, then content of intervention -- were nested (in the statistical sense) or crossed. It turns out that the “review” section groups them by design with intervention nested within; And then the discussion covers them again, but this time by intervention (all designs). The material is readable and accessible, when actually reading the two sections. This “heads up” could be less confusing if it dispensed with the “first, then” and just said the “The final seven studies were grouped according to the study design (RCT or prospective cohort) and the topic of the described intervention: ‘probability presentation’ (22, 23 ), ‘risk framing’ (24, 25, 26, 27), and ‘risk interpretation’ (28).”  **Response**  Edited accordingly |
| **24** | Lines 216 to 219. There are three instances where an extra “9” has been inserted.  **Response**  Edited accordingly |
| **25** | Line 216. The list might be changed from “…also perceived the risk to health to be higher (9p<0.0001), benefit to be lower (9 p=0.03), and were…” to ““…also perceived the risk to health to be higher (9p<0.0001) and the benefit to be lower (9 p=0.03), and were…”  **Response**  Edited accordingly |
| **26** | Line 232. There is an extra “,” in the “p <” parenthesis. I think this indicates that you went through and removed them all. Sorry for my contradictory advice.  **Response**  Edited accordingly |
| **27** | P 12, line 260. Could you add a comma after “pain” in “trial testing two drugs for post-operative pain one a standard treatment and the other”?  **Response**  Edited accordingly |
| **28** | Line 261. There is an orphan “t”.  **Response**  Edited accordingly |
| **29** | Line 265. Comma after “risk reduction”  **Response**  Edited accordingly |
| **30** | Line 266. Change “parent show” to “parents who”. Autocorrect sometimes errs.  **Response**  Edited accordingly |
| **31** | Line 271. Add “to be” before “more likely to agree”.  **Response**  Edited accordingly |
| **32** | The Tait study seemed to manipulate whether disadvantageous information was presented or hidden. Some of the other studies manipulate how disadvantageous (and advantageous) info is described, framed, but they present essentially the same in every condition. Although you touch on this briefly in the discussion (pp 17-18), it seems to be a very important distinction between the kinds of manipulation in these studies. The implications of the findings are different. When you find the (obvious) that hiding the disadvantages makes people more likely to choose an option, that informs the researcher/sponsor’s decision whether to lie and obfuscate in a propagandistic marketing manner. When you find that the same information, framed differently, influences option choice, that informs the researcher/sponsor’s decision whether to “nudge” in a libertarian paternalistic manner <https://en.wikipedia.org/wiki/Libertarian_paternalism>.  **Response**  The reviewer raises an interesting point relating to how participants decisions could be nudged in different directions depending on what or how information is presented to them. We have added a short extension to the final sentence in that paragraph to flag the potential problems of ‘nudging’. See page 18 lines 406-408. |
| **33** | P 14, line 299. “Participants were presented with information in one of six combinations in a pre-specified order.” First, you might put “the” before “six,” since you have already described the 2 (order) by 3 (content) design. But I thought that each person got only one condition? What is the function of “in a prespecified order” in this sentence? If it is about the two sequences, you have already incorporated it. Is there a different order? Of course there is a “procedure” that has a lot of steps in order: meet, qualify, consent, instruct, and so on. Usually that need not be said. Could you drop “in a pre-specified order”?  **Response**  Edited accordingly |
| **34** | Line 308. You could drop the conversational “When it came to presentation preferences” and the reader would still know exactly what you are talking about.  **Response**  Edited to amend conversational style but still retain explanatory introduction. |
| **35** | Line 318 add “and” between “risk” and “benefit”.  **Response**  Edited accordingly |
| **36** | Line 319. Add “noted only” after 27%.  **Response**  Edited accordingly |
| **37** | P 18, line 408, change punctuation and one word to “An analysis of PILs used in clinical trials by Gillies et al (2011) found that explaining trial processes, presenting probabilities, and expressing values were consistently poor across all PILs when assessed using an informed consent evaluation instrument”.  **Response**  Edited accordingly |
| **38** | P 19. Lines 448-449, change “indicates that there is as yet not clear optimal method for improving participant understanding, or clear consensus on how” to “indicates that there is as yet not a clear optimal method for improving participant understanding, nor a clear consensus on how”  **Response**  Edited accordingly |
| **39** | Line 451, change “within trials” to “about trials” or “during trials”? “within” evokes my ANOVA framework.  **Response**  Edited accordingly |
